# Clinico-genomic study reveals association of dengue virus genome high frequency mutations with dengue disease severity

**DOI:** 10.1038/s41598-025-00462-z

**Published:** 2025-05-28

**Authors:** Varsha Ravi, Md Imran, Kriti Khare, Pallavi Mishra, Ramakant Mohite, Kanika, Md Abuzar Khan, Aparna Swaminathan, Aanchal Yadav, Sristi Sinha, Richa Shukla, Partha Chattopadhyay, Jyoti Soni, Ranjeet Maurya, Tavpritesh Sethi, Bansidhar Tarai, Sandeep Budhiraja, Rajesh Pandey

**Affiliations:** 1 INtegrative GENomics of HOst-PathogEn (INGEN-HOPE) Laboratory, Division of Immunology and Infectious Disease Biology, CSIR-Institute of Genomics and Integrative Biology (CSIR-IGIB), North Campus, Near Jubilee Hall, Mall Road, New Delhi, Delhi 110017 India; 2 Indraprastha Institute of Information Technology, New Delhi, Delhi 110020 India; 3 Academy of Scientific and Innovative Research (AcSIR), Ghaziabad, 201002 India; 4 Max Super Speciality Hospital (A Unit of Devki Devi Foundation), Max Healthcare, New Delhi, Delhi 110017 India

**Keywords:** Mutation analysis, Disease severity, Dengue, Leukopenia, Thrombocytopenia, Immunology, Microbiology, Pathogenesis

## Abstract

Transmission of the dengue virus (DENV) places a huge burden on public health in several endemic regions. Like other RNA viruses, mutations in the DENV genome greatly governs its virulence, transmissibility, and interaction with the host immune system. Present study focuses on integrated analysis of mutation and clinical data accompanied at the onset of dengue fever. The findings from the associated clinical data with the variants of the DENV are critical for early detection of the disease and understanding the disease progression. RNA was isolated from the 1310 serum samples collected from the NS1-antigen positive dengue patients. Serotyping reveals that DENV-2 was predominant in circulation. The genome of 1305 DENV-2 was sequenced using Oxford Nanopore Technology and Illumina platforms. A total of 1023 DENV-2 demonstrated > 50% genome coverage. Mutation analysis across the 1023 DENV-2 genomes yielded a total of 2667 mutations including 627 non-synonymous and 2040 synonymous mutations. We observed a notable over-representation of synonymous mutations in *prM* and *ancC* genes while a higher occurrence of non-synonymous mutations was found in ancC, prM, and M proteins. Comparison of mutation frequency between mild and severe demonstrates higher mutation frequency in severe phenotype. Moreover, we observed a total of 56 significant mutations including 23 in severe, 17 in moderate and 16 in mild. The E-protein having non-synonymous mutations were docked with DC-SIGN with lower binding energy (ΔG = − 11.9 kcal/mol) for severe as compared to mild (ΔG = − 13.5 kcal/mol), suggesting lesser affinity of E-protein and DC-SIGN in case of severe as compared to the mild. We have identified the core set of high frequency mutations significantly associated with distinct dengue disease severity viz., mild, moderate and severe. Furthermore, in-silico protein modelling and docking studies demonstrate the potential functional role of the non-synonymous mutations identified across E-protein in severe dengue.

**Supplementary Information:**

The online version contains supplementary material available at 10.1038/s41598-025-00462-z.

## Introduction

Dengue, spread by the *Aedes aegypti* mosquito, is a significant public health concern, particularly in tropical and subtropical regions^1^. The DENV belongs to the Flaviviridae family and has four serotypes (1–4) responsible for the disease. DENV infection often manifests without symptoms, but when symptoms appear, they usually begin 3–14 days post-infection and include fever, headache, muscle and joint pain, and a distinctive skin rash. In a minority of cases, the illness can progress to severe dengue, a potentially fatal condition marked by bleeding, low platelet count, and plasma leakage^2^. This contributes to the 100–400 million dengue cases reported annually^3^. The highest number of global cases was recorded in 2023, with 6.5 million cases and over 7300 deaths worldwide. India reported its highest dengue cases in 2022, with 233,251 cases and 303 deaths that year^4,5^. The growing public health impact of dengue, with increasingly frequent and intense outbreaks, underscores the necessity for enhanced genomic surveillance and control measures^6^.

The DENV genome consists of approximately 11 kilobases of single-stranded positive-sense RNA with 5′ untranslated region (5’UTR), coding region, and 3′ untranslated region (3′UTR). The coding region consists of genes for both structural (capsid, envelope, and membrane) and non-structural (*NS1, NS2A, NS2B, NS3, NS4A, NS4B*, and *NS5*) genes that are essential for viral replication and pathogenesis^7^. Monitoring the genetic variations of the DENV through genomic surveillance is essential to track the emergence and spread of different serotypes and genotypes, which can result in plausible severe outbreaks. Additionally, a complete blood count (CBC) plays a crucial role in diagnosing and monitoring DENV infection by providing important information on blood cell dynamics, which can be modulated by the virus. Two significant hematological abnormalities associated with DENV infection are leukopenia and thrombocytopenia, which serve as crucial indicators of disease severity and progression. Thrombocytopenia and leukopenia observed in 90% and 76% of the cases, respectively, are among the significant hematological indicators of dengue disease severity^8^. This decrease in leukocytes and thrombocytes compromises the immune system’s ability to combat secondary infections^9^. Additionally, the percentage of lymphocytes predicts the hospital stay; the higher the lymphocyte count, the faster the recovery^8^. Therefore, leukopenia and thrombocytopenia can be considered critical markers for monitoring the disease progression in DENV infection.

India has witnessed a notable increase in dengue cases, with approximately 0.8 million cases recorded from 2017 to 2022, including around 1000 deaths. The post-monsoon period has consistently shown a spike in cases, aligning with the typical seasonal pattern of dengue transmission in the region. The DENV-2 serotype, particularly the cosmopolitan genotype, has been linked to outbreaks across multiple states in India. Reports indicate that DENV-2 was associated with significant outbreaks in northern cities such as Delhi, Lucknow, and Gwalior during this period^10,11^. The genetic analysis of circulating strains shows that cosmopolitan genotype has become increasingly prevalent since its emergence in the region. The predominance of DENV-2 serotype and its cosmopolitan genotype in India from 2017 reflects a complex interplay of rising case numbers, population immunity dynamics, environmental factors suitable for *Aedes* mosquito breeding, combined with urbanization and climate change, have facilitated higher transmission rates of dengue virus^12^. Mutations in the DENV genome can significantly affect its virulence, transmissibility, and interaction with the host immune system. There are few earlier reports which investigated the role of mutations in the DENV genome in disease severity^13–16^. Briefly, Hapuarachchi et al.^13^ observed an isoleucine to valine substitution at position 322 in the EDIII domain of the E gene of DENV-2 manifesting dengue fever (DF) and dengue haemorrhagic fever (DHF) in the patient. The envelope protein, essential for host cell entry, is a major determinant of virulence and is subject to immune selection pressure^17^. This pressure can lead to mutations that facilitate immune escape, thereby enhancing the virus’s ability to evade the host immune response^12^. Another study highlights the presence of the G605V mutation in the NS5 region of DENV-2, which was predominantly linked with more virulence and severe dengue conditions in mouse models^15^. Additionally, a study highlighted the presence of two silent mutations, T7812G (G81G) and C9420A (A617A) in the NS5 region, known to play an essential role in the viral replication^18^. A mutation reported in the NS4 regions linked to impaired replication efficiency of the DENV genome, possibly resulting in milder clinical outcomes due to the lower viral titer^13^. Furthermore. Chan et al.^19^ observed a strong correlation between the T164S mutation and increased disease severity in DENV infection. Importantly, through the literature survey, we found specific mutations associated with primary and secondary infections. Maria et al.^20^ identified L124F mutation in the NS1 protein associated with Dengue Fever in primary infection. Additionally, E71A, D154N, and G330D mutations in the E-protein were associated with dengue with warning signs and severe dengue during secondary infection. These findings underscore the importance of continuous genomic surveillance to monitor and understand the implications of these mutations for DENV transmission, pathogenicity, surveillance, and severity progression. Having the background where the role of few specific mutations across DENV genomes have been established in severe dengue, a large-scale clinical genomic surveillance of DENV focusing on the analysis of genome variability vis-à-vis disease severity is essential.

Therefore, we aimed to investigate whether DENV genome mutations can be plausible indicators for dengue disease severity. Towards this, we have used the samples collected from the hospital admitted dengue patients during the major outbreak season in Delhi, India in 2022. The present study aims to comprehensively analyze the mutations across the in-house sequenced DENV genomes vis-à-vis the analysis of hematological parameters across the patients categorized into distinct disease severity, viz., mild, moderate, and severe. We observed the predominance of DENV-2 serotype in circulation, followed by co-circulating DENV-3 serotype, indicating their possible role in co-infection during dengue pathogenesis. Integrative genomic mutation analysis revealed the association between high-frequency mutations and clinical parameters, including leukopenia and thrombocytopenia, suggesting their critical involvement in driving or modulating dengue severity.

## Materials and methods

### Sample collection and processing, RNA isolation, and DENV-serotype detection

Serum samples from NS1-Ag-positive patients were collected from Max Super Speciality Hospital from August 2022 to November 2022. The demographic characteristics such as age, median age and gender of the patients included in the study is in Table 1. The serum samples received on a daily basis were separated and stored at − 80 °C until processed for RNA isolation. For each sample, a unique ID was assigned. RNA was extracted from 150 µl of serum using the QIAamp Viral RNA Mini Kit (Qiagen, Cat No. 52906). The quantity and quality of the RNA were determined using NanoDrop. Using RNA as a template, dengue serotype-specific qRT-PCR was performed. For the detection of DENV-serotypes, DENV serotyping kit (Genes2me; cat. No. G2M705021) was used and the protocol was followed as per the manufacturer’s instructions.

**Table 1:** Summary of demographic characteristics of the 1056 dengue positive patients.

Age distribution (years)	Female	Male
0–15	86	165
16–30	199	240
31–45	102	152
46–60	37	34
> 61	20	21
Total	444 (42%)	612 (58%)
Min (Age)	0	0
Max (Age)	82	84
Median (Age)	27	25

### Whole genome sequencing of DENV-2

We have sequenced 1305 DENV-2 genomes using Oxford Nanopore Technology (ONT) and Illumina platform. Specifically, 1013 DENV-2 genomes were sequenced using ONT whereas 292 DENV-2 genomes were sequenced using Illumina platform.

#### Nanopore sequencing

DENV-2-positive RNA samples were reverse transcribed into cDNA using the LunaScript RT Supermix (New England Biolabs, Cat. No. E3010L). This cDNA served as a template for the amplification of DENV-2 genomes. Amplification was performed using 39 pairs of overlapping PCR primers and the Q5 High Fidelity 2X Master Mix (New England Biolabs, Cat. No. M0494L). To amplify the DENV-2 genome, we have used primers adopted from Stubbs et al.^21^ 2020 and optimized in our lab for its optimal working. The sequence of the primer is provided in the Supplementary Table 1. The primers utilized work on multiplex PCR tilling approach and produced 400 base pairs overlapping amplicons having the average overlap of 128 bps between the amplicons. The 39 pairs of primers used could produce 39 overlapping amplicons that together cover the complete DENV genome. The PCR reactions were divided into two pools, each containing either an odd or even set of primers. Following PCR amplification, the two pools were combined and purified using Ampure XP (AXP) beads. The purified PCR products underwent end repair using the NEBNext Ultra II End Repair/dA-Tailing Module (New England Biolabs, Cat. No. E7546L). Unique barcodes were then ligated to the end-prepped PCR products using the Rapid Barcode Kit (SQK-RBK110-96) and Native Barcoding Expansion kits (Exp-NBD114, SQK-LSK109, and Exp-NBD196). We have used R.9.4.1 flow cells. The barcoded samples were pooled and purified again using AXP beads. Finally, adapters were ligated to the barcoded, purified products, which were then loaded onto an Mk1C device for sequencing.

#### Validation across R.9 and R10 flow cells

We also validated the insights from the Dengue genomes sequenced using R.9.4.1 flow cells by re-sequenced a random subset of 50 samples from the study using R10.4.1 flow cells and sequenced using a GridION device (ONT) device, followed by high-quality DENV-2 genome analysis. Following the completion of the sequencing runs on the GridION device, the raw ONT reads were transferred to high-performance computing (HPC). Briefly, raw reads were basecalled and demultiplexed through Guppy basecaller and barcoder with a phred Q-score quality of > 9. The demultiplexed high-quality passed reads were mapped to the DENV-2 reference (NC_001474) genome using minimap2. Additionally, primer trimming was carried out using align trim. Further, artic_mask was used to mask the low-quality reads and taken as ‘coverage_mask’ files. Primer depleted high-quality DENV-2 reads were taken for variant calling through Clair3. Post-VCF filtering was carried out using bcftools by removing the SNVs having genotype quality less than 3 and depth less than 20 as ‘fail_vcf’ and taking genotype quality greater than 3 and depth greater than 20 as ‘pass_vcf’. Lastly, consensus FASTA was created by masking the positions of ‘fail_vcf’ and ‘coverage_mask’ and implementing the SNVs from ‘pass_vcf’ to the DENV-2 reference genome using bcftools.

#### Illumina sequencing

Library preparation for whole genome sequencing for DENV-2 was carried out using the Illumina COVIDSeq protocol (Cat. No. 20043675, reference guide: 1,000,000,126,053 v04). The protocol began with cDNA preparation using isolated RNA from the DENV-2 samples. The viral genome was then amplified through two separate PCR reactions. The resulting pooled amplicons were subjected to tagmentation, which fragmented and tagged them with adapter sequences. This was followed by a cleanup step to remove post-tagmentation residues and a second round of PCR amplification was performed to ligate index adapters. The indexed amplicons were subsequently pooled and purified using AXP beads.

Quantification of the pooled library was performed using the Qubit dsDNA HS Assay Kit (Cat. No. Q32854). The libraries were first normalized to 4 nM, followed by the final 650 pm according to the NextSeq 2000 System Denature and Dilute Libraries Guide (Illumina, Document # protocol 1,000,000,126,053 v04). A total of 20 µl 650 pm of the final pooled normalized library was sequenced in the NextSeq 1000/2000 P2 flow cell.

### Genome sequence analysis

#### Nanopore sequencing

The raw ONT reads from Mk1C were transferred to High-Performance Computing (HPC) and analyzed through the ARTIC end-to-end pipeline^22^ until variant calling and consensus FASTA creation. Raw reads were basecalled and demultiplexed through Guppy with a phred-score quality of > 7. The Guppy utilizes “fast” accuracy algorithm, enabling efficient basecalling and barcode demultiplexing. The demultiplexed files were aligned to the DENV-2 reference (NC_001474) with minimap2^23^, and the depth and breadth of coverage was determined (Supplementary Table 2). The reference genome used is a wild type of DENV Serotype-2 and is not specific to India or any particular genotype. Further, variant calling was done for the aligned reads using indexed Fast5 files, quality-controlled FASTQ reads through Nanopolish^24^ and consensus FASTA was created using bcftools^25^.

#### Illumina sequencing

The raw reads from Illumina NextSeq2000 were transferred to in-house High-Performance Computing (HPC). Initially all the FASTQ files are proceeded for QC using FASTQC with phred score cut-off greater than 20 (Babraham Bioinformatics—FastQC, A Quality Control tool for High Throughput Sequence Data) and low quality reads were removed using trimmomatic^26^. The filtered reads were aligned using bowtie2^27^ and BAM files were generated. Further, variant calling file and consensus fasta were generated using bcftools^25^.

### Phylogenetic analysis

The DENV-2 virus genomes demonstrated > 50% genome coverage (n = 1023) were used for the calling of genotype information and construction of a phylogenetic tree. Genome coverage of > 50% was selected as minimum because of platform to share the data in public domain through GISAID. Firstly, multiple sequence alignment was done using MAFFT (v7.475)^28^. The alignment was manually trimmed and genotyping information was generated using the Genome Detective tool (v3.46)^29^. Further, a phylogenetic tree was constructed using IQ-TREE employing the maximum likelihood method^30^. The phylogenetic tree was visualized with iTOL software^31^.

### Mutation segregation and generation of mutation clusters

To capture and visualize the trend in the data, we have generated mutation/variation clusters based on the frequency. The mutations observed at different DENV genomic positions have different frequencies, ranging from zero to 100%. We have generated five mutation clusters spanning a frequency window of 20% gap. This approach builds on our previous published SARS-CoV-2 investigation, wherein we compared low frequency mutations (mutations obtained from less than 10% of the patients) with high frequency mutations (mutations obtained from greater than 45% of the patients)^32^. Similarly, the present analysis involved segregating mutation data into five distinct mutation clusters viz. cluster-A, cluster B, cluster C, cluster D and cluster E. To generate the mutation cluster, a methodological approach utilizing data filtering and clustering was implemented. Mutations having differences of frequency up to 20% across the mild, moderate and severe at particular genomic position fall within a cluster. For instance, mutations falling within the frequency range of 0–20% were assigned to the cluster—Cluster-E. This filtering process ensured that all severity categories exhibit mutation frequencies falling within the designated cluster range. Following this classification, mutations were grouped into clusters spanning the frequency intervals of 0–20, 21–40, 41–60, 61–80, and 81–100%.

### Clinical data segregation and statistical analysis

Statistical analysis for clinical parameters was performed using the Mann–Whitney U test in GraphPad prism. The clinical data was summarized using descriptive statistics. Continuous variables were depicted by their median, while categorical variables were expressed as percentages (n, %). Significance was determined at a *p*-value of < 0.05.

### Dengue severity classification

For our routine clinical DENV genomic surveillance, along with the serum samples of the NS1 positive dengue patients from the hospital partner, we also get the clinical details/symptoms of the patients, including detailed blood profile. The clinical parameters include various physiological and laboratory measurements that provide insights about the patients’ health condition and reflect degree of disease severity. Key hematological parameters such as platelet count, total leukocyte count (TLC) commonly monitored in DENV infection particularly in severe cases associated with dengue hemorrhagic fever (DHF) or dengue shock syndrome (DSS)^33^. For our study, out of 1310 dengue patients, we have received clinical data for the 1056 patients. This clinical data has been used for severity subgroup classification for the patients included in this study. Towards that, initially, we referenced the WHO guidelines (WHO 2009) to classify dengue severity, which includes categories such as dengue without warning signs, dengue with warning signs, and severe dengue. Interestingly, our patient classification into disease severity aligns with WHO classifications to a greater extent, except that none of the patients in our study exhibited severe bleeding, organ failure, or abnormal liver parameters. According to the WHO dengue classification and management scheme, these symptoms are essential for classifying severe dengue.

In the present study, patients falling into the criteria for dengue without warning signs and had normal platelet and leukocyte counts were classified as mild cases. Patients having dengue with warning signs were further divided into two groups: (1) Patients with normal platelet counts but with leukopenia were grouped as moderate cases whereas; (2) the patients with both thrombocytopenia and leukopenia were categorized as severe cases^34^. Although our patients did not fit neatly into the WHO-defined severity categories, we used these classifications—mild, moderate, and severe—for analytical purposes to ensure consistency and clarity in our data interpretation as follow:

- *Mild (n* = *317, Dengue without warning signs)*: Patients with normal platelet and leukocyte counts (no thrombocytopenia or leukopenia). A total of 317 patients demonstrated normal TLC and normal platelet count.
- *Moderate (n* = *222, Dengue with warning signs):* Patients with normal platelet counts but decreased leukocyte counts (leukopenia without thrombocytopenia). A total of 222 patients were having decreased TLC but normal platelet count was categorized as moderate.
- *Severe (n* = *185)*: Patients with both low platelet counts (thrombocytopenia) and decreased leukocyte counts (leukopenia). We have 185 patients with both decreased TLC and platelet count.

Using the above criteria, we have classified 724 patients in distinct diseases severity ranging from mild to severe for conducting the study of association mutations in DENV genome vis-à-vis disease severity.

### Modelling and docking analysis of E-protein with DC-SIGN

Considering that E-protein is the primary point of entry into the host, particularly interacting with the host’s DC-SIGN receptor, protein–protein docking between these two proteins were performed. Before performing protein–protein docking, the model of E-protein was generated. To find a suitable template for generating the model of E-protein, fasta sequence of the wild type E-protein was retrieved from the NCBI database (NP_739583) and a blastp search was executed against the Protein Data Bank (PDB) database^35^. The blast search yielded a potential template. We have listed five top potential templates in Table 2. Among these, PDB id:7KV8 was selected as template for generating the model of E-protein as it exhibited good resolution, and high query cover. Further, using MODELLER software^36^, the E-protein model was generated. The modelled protein’s accuracy was assessed by the lowest DOPE score (Discrete Optimised Protein Energy), comparing it with the original structure and obtaining the root-mean Square Deviation (RMSD), which was found to be 0.1 and the disallowed region in the Ramachandran plot was 0.15. To assess the impact on the severity level, non-synonymous mutations observe in the E-protein belonging to the cluster-A (80–100% frequency) vis-à-vis distinct disease severity such as mild, moderate and severe were introduced in to the E-protein model using PyMol. Additionally, the structure for DC-SIGN downloaded directly from PDB (PDBid:1SL4). Furthermore, protein–protein docking done using the HADDOCK webserver^37^ with active residues of 300 to 394 for E-protein and 340 to 370 residues for DC-SIGN, respectively. Interaction between E-DC-SIGN examined using PDBsum and further analyzed through DIMPLOT of LIGPLOT^38^.

**Table 2:** Possible templates for E-protein.

PDBid	Experiment	Resolution Å	Uniprotid
7KV8	Electron Microscopy	2.5	P18356
6ZQU	Electron Microscopy	3.1	P29990
7CTH	Electron Microscopy	3.5	P14340
3J27	Electron Microscopy	3.6	P14340
4UIF	Electron Microscopy	6.5	EOWXJ2
1P58	Electron Microscopy	9.5	P14337

## Results

### DENV-2 was the predominant serotype in circulation

**Fig. 1:**
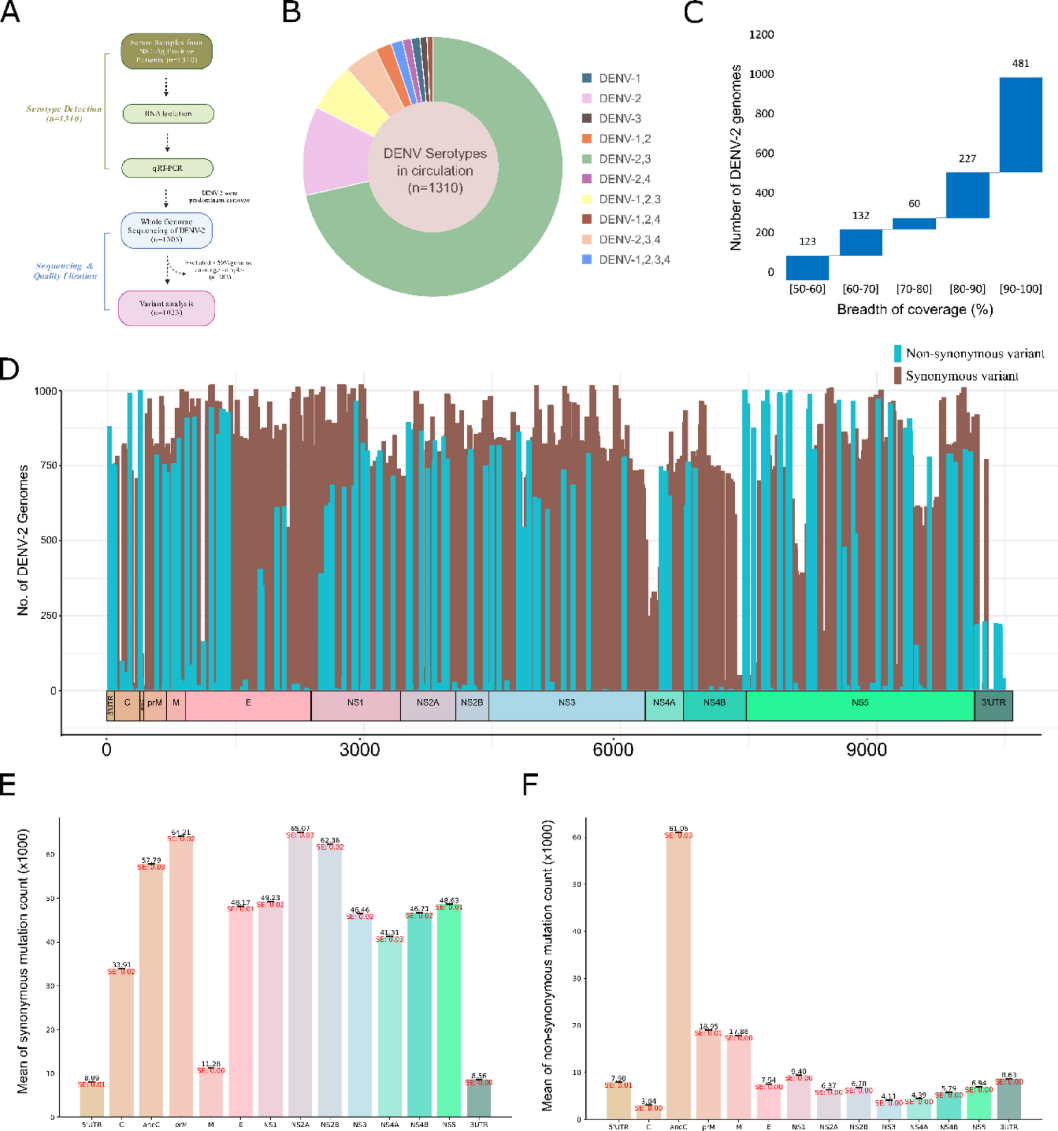
DENV serotype distribution and mutational dynamics across the DENV-2 genomes. (**A**) Overview of the experimental design, including RNA isolation from serum samples, followed by qRT-PCR, whole genome sequencing, and variant analysis. (**B**) The sunburst plot represents the distribution of DENV serotypes in circulation from August 2022 to November 2022 in Delhi, India. (**C**) Bar graph showing the range of breadth of coverage across the number of DENV-2 genomes sequenced. (**D**) Line-range plot depicting the mutation spectrum across the DENV-2 genomes. The length of each line indicates the number of samples in which genomic mutations were detected at that particular position. (**E–F**) Bar chart representing means of gene length normalized count, (**E**) synonymous, and (**F**) non-synonymous mutation counts (× 1000) across the DENV-2 genomes.

RNA isolated from the 1310 serum samples collected from NS1-antigen-confirmed dengue patients was used for serotype-specific qRT-PCR (Fig. 1A). Out of 1310, 153 were found positive for only DENV-2 (Table 3). Additionally, n = 3 and n = 2 were identified as only DENV-1 and DENV-3, respectively. Importantly, mixed infections of more than one serotype were also observed. Figure 1B represents the distribution of all singular or mixed serotypes detected in our dataset. Briefly, for 984 cases, in addition to the DENV-2, DENV-3 signals were also detected, indicating the DENV-2 and DENV-3 coinfection. Similarly, for n = 13 and n = 3, DENV-2 was co-infected with DENV-1 and DENV-4, respectively. For n = 84, DENV-1, DENV-2, and DENV-3 signals were recorded, whereas for n = 58, DENV-2, DENV-3, and DENV-4 were observed. For n = 9, all four serotypes were identified. In summary, out of 1310, 1305 were DENV-2 positive; however, in n = 1137, in addition to the DENV-2 serotype, DENV-3 was also detected. It is evident that during the dengue outbreak from August 2022 to November 2022 in New Delhi, India, DENV-2 was the predominant serotype, and DENV-3 was the major co-infecting serotype in circulation. We have inspected average Ct-values of Mild, Moderate and Severe to capture the potential difference in the viral load of patients depicting differential disease phenotype. The average Ct value across mild (20.20), moderate (21.30) and severe (20.60) was similar indicating the similar viral load across them. This finding indicates the role of genomic mutation within the DENV-2 population in differential severity rather than the overall viral load, emphasizing the crucial need to identify mutation hotspots in severe DENV through whole genome sequencing.

**Table 3:** Distribution of DENV Serotypes (n = 1310) in Circulation.

Serotypes	Number of samples	Percentage (%)
DENV-1	3	0.23
DENV-2	153	11.68
DENV-3	2	0.15
DENV-1, DENV-2	13	0.99
DENV-2, DENV-3	984	75.11
DENV-2, DENV-4	3	0.23
DENV-1, DENV-2, DENV-3	84	6.41
DENV-1, DENV-2, DENV-4	1	0.08
DENV-2, DENV-3, DENV-4	58	4.43
DENV-1, DENV-2, DENV-3, DENV-4	9	0.69

### Mutation dynamics across the DENV-2 genomes

A total of 1305 DENV-2 genomes were sequenced using Oxford Nanopore Technology and Illumina sequencing. We have generated significantly higher numbers of reads with an average high quality passed reads of 429,246 per Dengue virus genome. The high quality passed reads were mapped to the DENV-2 reference genome and coverage depth and breadth was determined. We achieved an average coverage depth of 812X for the DENV-2 population. Out of 1305, 282 DENV-2 genomes demonstrated less than 50% genome coverage breadth, with 1023 DENV-2 genomes of higher genome coverage with respect to the reference genome. Precisely, out of 1023, for the 481 (47.12%) DENV-2 genomes, ≥ 90 to ≤ 100% genome coverage was achieved (Fig. 1C). Similarly, for the 227 (22.19%) DENV-2 genome, genome coverage was in the range of ≥ 80 to ≤ 90%. For the 60 (5.77%), 132 (12.90%) and 123 (12.02%) DENV-2 genomes, the breadth of genome coverage was in the range of ≥ 70 to ≤ 80%, ≥ 60 to ≤ 70% and ≥ 50 to ≤ 60%, respectively. The variation in genome coverage may be attributed to the direct sequencing of genomes from the clinical samples. It could also reflect differences in the availability and integrity of the DENV RNA genome within the clinical serum samples vis-à-vis its journey from hospital collection to reaching the research lab for sequencing. Thus, 1023 DENV-2 genomes subjected to mutation analysis had an average genome coverage of 82.36%. Mutation analysis across the 1023 DENV-2 genomes yielded a total of 2667 mutations. The mutation spectrum across these DENV-2 genomes shows the positions of the mutations.

Further, we have classified total genomic variations (mutations) broadly into two categories, viz., synonymous and non-synonymous mutations. Out of 2667 mutations, 627 were non-synonymous mutations, and the remaining 2040 were synonymous (Fig. 1D). Gene-wise distribution of mutations reveals the high number of mutations in the *NS5* gene, whereas the least number of mutations were noticed in the 5′UTR. Further, we did gene normalization to observe the relative abundance of mutations with respect to the gene length across the DENV-2 genomes. Our analysis of the gene length normalized count of synonymous mutations across the DENV-2 genomes indicated the over-representation of synonymous mutations in the *prM* and *ancC* amongst the structural genes and *NS2A* and *NS2B* amongst the non-structural genes. The high occurrence of synonymous mutations could potentially alter the transcript of the genes. Similarly, the over-representation of non-synonymous mutations was observed in anC, prM, and M proteins (Fig. 1E). The high mutation rate for the non-synonymous mutations in the ancC, prM, and M proteins could potentially alter the protein sequence and affect its functions (Fig. 1F). Virulence and survival of the virus inside the host are accompanied by the successful replication of the viral genome as well as the efficient functioning of viral structural and non-structural proteins.

It was imperative to look for the domain-wise distribution of non-synonymous mutations in the DENV-2 genome. We observed many high-frequency (> 80%) non-synonymous mutations in various domains of major DENV-2 proteins (Supplementary Table 3). Two high-frequency mutations, i.e., S101T and L108M, were observed in the ancC domain. These amino acid substitutions were within the same polarity type, i.e., both Serine and Threonine are hydrophilic polar uncharged amino acids, and the Leucine to Methionine substitution is also within the hydrophobic aliphatic polarity type. Additionally, various other high-frequency mutations were observed in important functional domains of DENV-2 proteins. The N-terminal domain of the prM protein, which is cleaved during the maturation and formation of M proteins, is observed with two high-frequency mutations at positions 82 and 83. M protein, on the other hand, had high-frequency mutations both in the ectodomain and the C-terminal transmembrane region. E protein, however, had multiple high frequency mutations in all of its domains, such as DI (two mutations), DII (three mutations), DIII (two mutations), and the transmembrane domain (three mutations), except the stem region, indicating potential modifications in viral assembly and entry into the host. Similarly, relevant mutations were observed in the non-structural proteins. We observed two mutations in the wing domain and the C-terminal domain of the NS1 protein. NS2A, on the other hand, only had one mutation in the transmembrane segment (TMS5) region. Interestingly, NS2A and NS4B proteins observed mutations in the connecting regions between functionally important domains, i.e., between α-2 and α-3 for NS2A and α-7 and α-8 for the NS4B protein. NS4B also harbored one mutation in the α5 region. Captivatingly, the NS3 protein had only three mutations in the N-terminal region, while no high frequency mutation was observed in NS4A. Another fascinating observation was the presence of multiple (five in the methyltransferase (MTase) domain, one in the linker region, and six in the RdRp domain) high frequency mutations in the NS5 DENV-2 protein, implicating important functional consequences in viral replication.

### Clinical data demonstrate high proportion of leukopenia and thrombocytopenia in patients

**Fig. 2:**
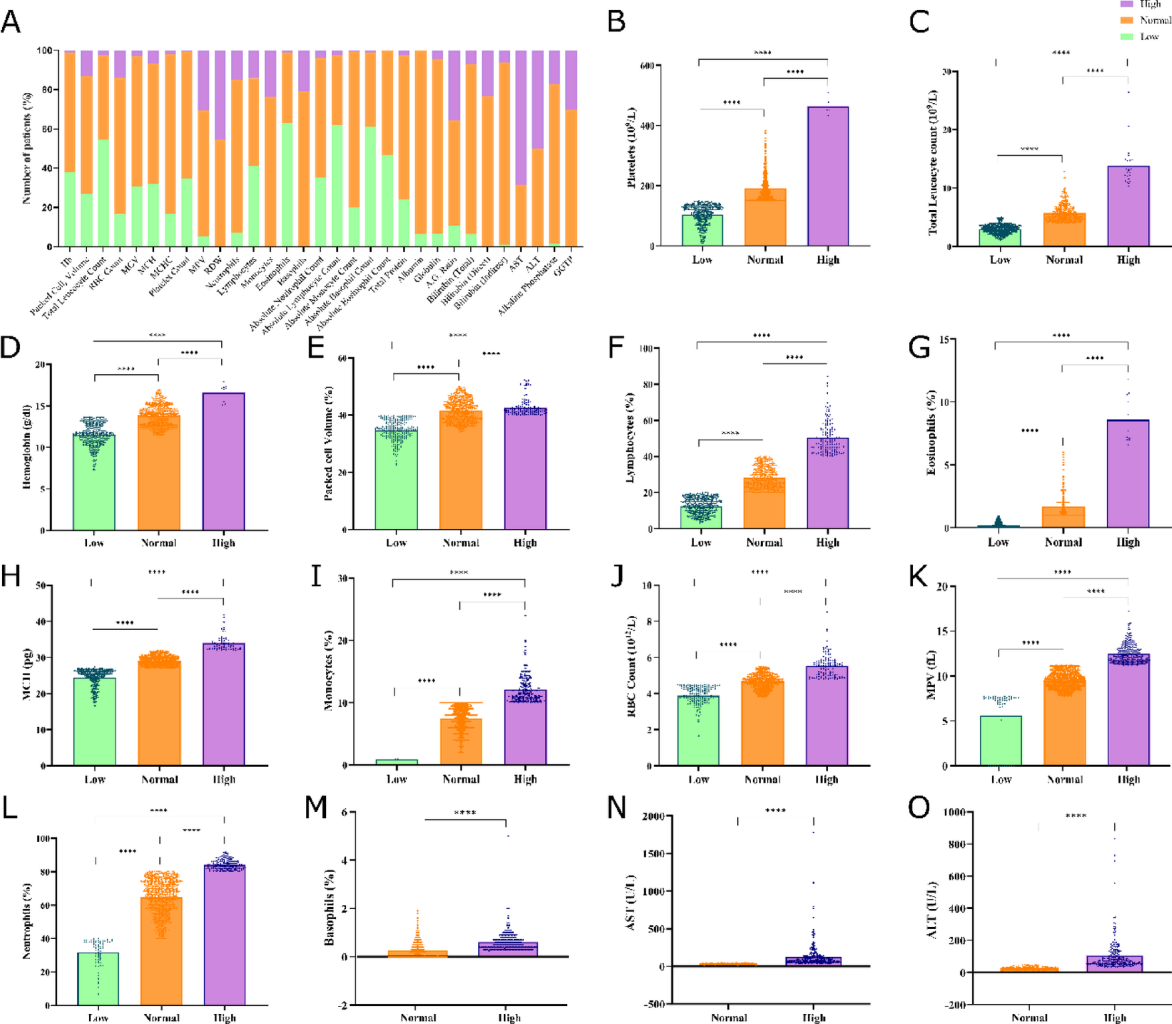
Clinical characteristics of dengue-positive individuals. (**A**) Stacked bar plot representing the percentage distribution of dengue patients across all the clinical parameters, categorized as low, normal, and high based on their clinical parameters derived from both complete blood count (CBC) and liver profile test (LFT) reports. (**B–O**) Sample-wise distribution of clinical parameters for (**B**) platelet count (10^9^/L), (**C**) total leucocyte count (TLC) (10^9^/L), (**D**) hemoglobin (g/dl), (**E**) packed cell volume or hematocrit (%), (**F**) lymphocytes (%), (**G**) eosinophils (%), (**H**) mean corpuscular hemoglobin or MCH (pg), (**I**) monocytes (%), (**J**) red blood cell or RBC count (10^12^/L), (**K**) mean platelet volume or MPV (fL), (**L**) neutrophils (%), (**M**) basophils (%), (**N**) aspartate aminotransferase or AST (U/L), and (**O**) alanine aminotransferase or ALT (U/L). The points above the bar represent samples that were categorized according to the range of each parameter: low: green, normal: orange, and high: purple.

By the onset of fever in DENV-infected patients, the disease may progress towards a distinct severity ranging from mild to severe. The progression of DENV infection towards severity is known to widely alter the complete blood count (CBC) of the patient. Therefore, the CBC accompanying the onset of dengue fever was comprehensively analyzed and we observed a wide range of deviation of the hematological parameter from the normal range. The normal range for each blood parameter as per WHO guidelines was set, and the observed hematological parameters were segregated as low, normal and high (Supplementary Table 4). We have the clinical data for hematological parameters inclusive of hemoglobin count (Hb), packed cell volume (PCV), total leukocyte count (TLC), mean corpuscular volume (MCV), mean corpuscular hemoglobin (MCH), mean corpuscular hemoglobin concentration (MCHC), mean platelet volume (MPV), red cell distribution width (RDW), neutrophils, lymphocytes, monocytes, eosinophils, and basophils for 1003 patients (Fig. 2A), whereas the platelet count for a total of 1032 patients. In our dataset, 54.6% (n = 548) patients had low TLC, whereas 34.9% (n = 361) patients manifested a low platelet count (Fig. 2B and C), indicating the development of leukopenia and thrombocytopenia, respectively. Moreover, the hematocrit rise was recorded in 12.8% (n = 129) of patients.

The other blood parameters (Fig. 2D–M), such as Hb count, were observed to be low in 38.1% (n = 382) patients, whereas the MCH and MCHC were recorded as low for 31.9% (n = 320) and 16.6% (n = 167) patients, respectively. Low Hb, MCH and MCHC counts indicate the development of anemia in dengue patients. Additionally, we had parameters associated with liver functions such as the level of AST (n = 377) and ALT (n = 378) in the blood of patients infected with the DENV. Our analysis indicates that the levels of AST and ALT were high in 68.4% (n = 258) and 49.7% (n = 188) patients, respectively (Fig. 2N and O). The AST and ALT levels were normal in the rest of the patients, whereas low levels of AST and ALT were observed in none of the patients in our dataset. The Mann–Whitney U test indicates that all deviations from the normal range, including normal to low or normal to high, were significant, indicating the significant changes in the blood parameter at the onset of dengue fever.

### Predominance of cosmopolitan genotype in DENV-2 serotype

**Fig. 3:**
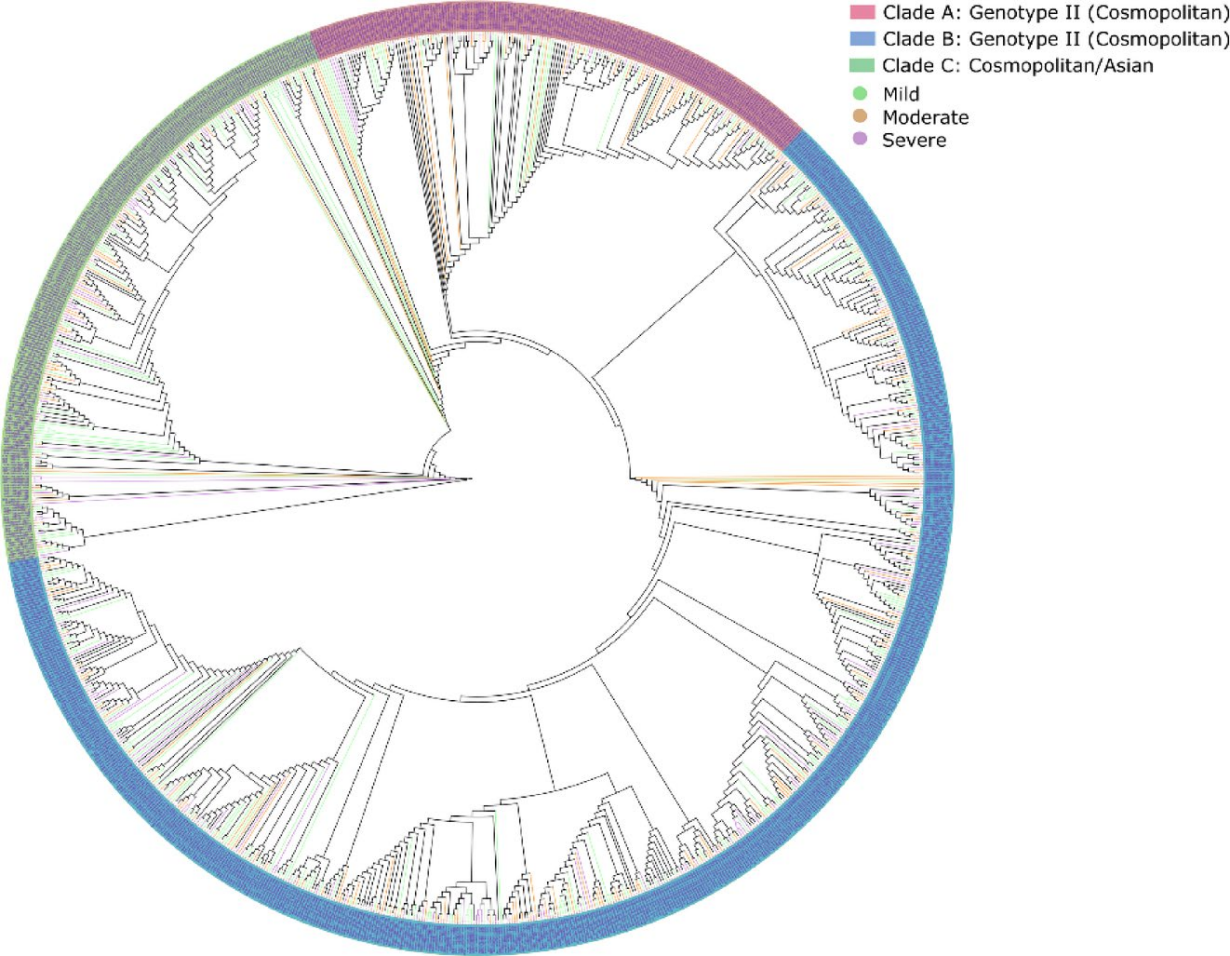
Phylogenetic analysis of 1023 DENV genomes and depiction of genotypes identified. The analysis revealed three clades of DENV-2 serotypes, where clade A and clade C represent cosmopolitan, while clade B is a mixture of both cosmopolitan and asian-I. The clades with different genotypes are labeled as mild (green), moderate (orange), and severe (purple).

Phylogenetic tree was constructed for 1023 DENV-2 genomes. The phylogenetic tree analysis identified three distinct clades namely A, B, and C. Clade A includes 616 genomes, of which 615 are classified as Cosmopolitan genotypes. Clade B is composed of 226 genomes, displaying a more balanced composition with 73 Cosmopolitan and 107 Asian I samples. Clade C contains 181 genomes, all of which belong to the Cosmopolitan genotype (Fig. 3). These findings highlight the predominance of the Cosmopolitan genotype in our dataset, comprising 85.22% of the total cases, with the Asian-I genotype representing 10.46% of the cases. Furthermore, the cosmopolitan genotype and Asian-I falling in to distinct disease severity is tabulated below (Table 4).

**Table 4:** Mutation distribution across the cosmopolitan-II and Asian-I genotypes belonging to mild, moderate and severe.

Severity	Total samples	Cosmopolitan type II	Percentage (%)
Mild	317	269	85.12
Moderate	222	193	86.93
Severe	185	151	81.62

Severity	Total samples	Asian I	Percentage (%)
Mild	317	37	11.70
Moderate	222	16	7.20
Severe	185	26	14.05

### Association of high frequency mutation with dengue disease severity

**Fig. 4:**
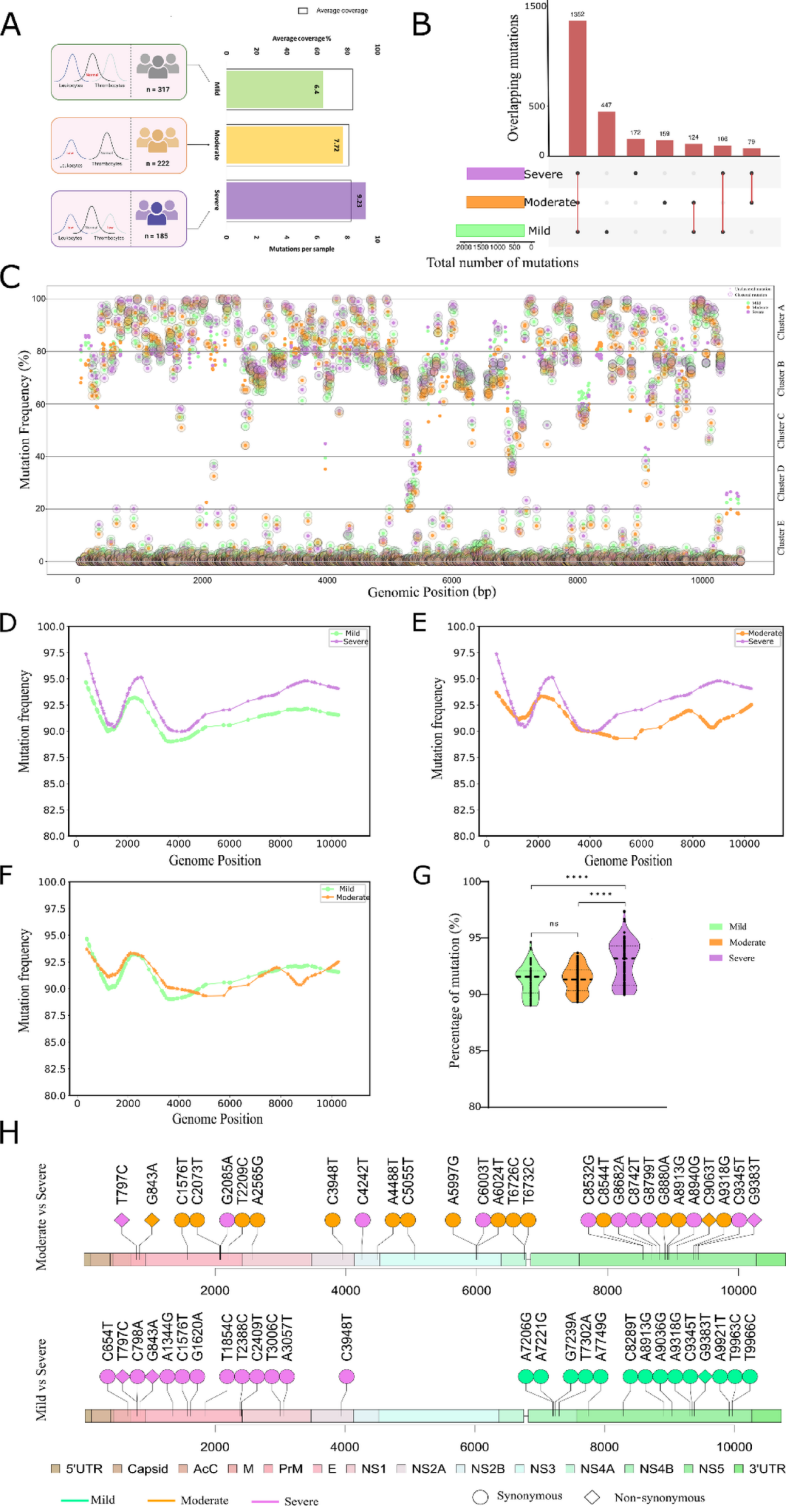
Association of high frequency mutation with distinct disease severities. (**A**) Graphical representation of dengue patients (n = 724), where mild (n = 317), moderate (n = 222), and severe (n = 185) were categorized based on the leukopenia and thrombocytopenia clinical parameters. The bars depict the average coverage and mutations per sample across mild, moderate, and severe. This figure was created using Biorender.com. (**B**) The upset plot shows the number of overlapping mutations across mild, moderate, and severe patients. (**C**) Scatter plot representing percentage of mutation frequency across the DENV-2 genomes. The dots within the circle depict mutation frequency within the cluster (clustered mutation), while only the dots represent mutation frequency outside the cluster (unclustered mutation). The color represents mild, moderate, and severe. (**D–F**) Line plots showing mutation frequency across the DENV-2 genome for Cluster A (80–100%) between three comparison groups: (**D**) mild vs. severe, (**E**) moderate vs. severe, and (**F**) mild vs moderate. (**G**) Violin plot showing significant differences between percentage of mutation and severity cohort (mild, moderate, and severe) for cluster A. One-way ANOVA test was used for the significant test (*p* value < 0.05). (**H**) Lollipop plot representing significant synonymous and non-synonymous mutations between 2 comparison groups, i.e., moderate versus severe (upper) and mild versus severe (lower), across the DENV-2 genomes.

DENV infection frequently manifests in development of leukopenia and thrombocytopenia in the patients. In our clinical data set, we had TLC and platelet count data for 724 patients. Based on our clinical data, the dengue patients (n = 724) were classified into three disease severity groups. DENV infection with normal TLC and platelet count were classified as mild (n = 317). DENV infection manifested by a low TLC count and normal platelet count was grouped as moderate (n = 222), whereas the dengue-positive patient manifested with both a low TLC count and a low platelet count was classified as severe (n = 185) (Fig. 4A). The mutations of mild, moderate and severe patients were analyzed separately to know the unique and common mutations between the groups. Delving deeper, the distribution of mutations across the severity group results in the appearance of 2029 mutations in the mild patients, 1714 mutations in the moderate, and 1709 mutations in the severe patients (Fig. 4B) (Supplementary Table 5).

Importantly, the relative count of mutations (mutation/sample) evinces a higher number of mutations exhibited by the severe patients (9.23 mutations/patient) as compared to the moderate (7.72 mutations/patient) and mild (6.40 mutations/patient). The observation of a relatively higher number of mutations per patient in a severe led us to compare the frequency of mutations across the viral genome amongst the severity subgroup. Therefore, with the objective of comparing the mutation frequency across the virus genome and amongst the disease severity subgroups, we have generated five mutation clusters ranging in mutation frequency from 81 to 100%, 61 to 80%, 41 to 60%, 21 to 40%, and 0 to 20%, in each cluster. For the sake of ease, the mutation clusters were designated as clusters A, B, C, D, and E, respectively (Fig. 4C). When we compare the frequency of mutation falling in cluster A between mild and severe, the severe patients exhibited high frequency as compared to the mild across the virus genomes (Fig. 4D). Additionally, with the exception of two genomic positions, the mutation trend had a high-frequency in the severe as compared to the moderate patients (Fig. 4E). However, similar comparisons between moderate and mild resulted in a mutation trend with similar frequency in both mild and moderate patients (Fig. 4F). The one-way ANOVA analysis indicated that mild vs. severe and moderate vs. severe are significant; however, mild vs. moderate is non-significant (Fig. 4G). The comparison of mutation frequency among the severity subgroups for clusters B, C, D, and E was not significant (Supplementary Figure 1), indicating the plausible role of high-frequency mutations (cluster A) in distinct disease severity ranging from mild to severe in DENV infection.

### Statistical analysis yielded mutations of significance across the severity subgroups

To further comprehend the significance of mutations associated with the severity subgroups, fisher’s exact test was performed along with phi-correlation coefficient analysis to understand the direction of mutations associated across mild, moderate, and severe, considering a *p*-value of < 0.05. We observed a total of 56 statistically significant mutations across the severity subgroups. Out of the 56 mutations, 23 mutations were significantly associated with the severe patients whereas 17 and 16 mutations were significantly associated with the moderate and mild patients, respectively. It is interesting and important to mention that, eight mutations were non-synonymous amongst which mild and severe had three mutations each whereas two were associated with the moderate patients (Fig. 4H). Out of eight mutations, five mutations were present in the NS5 (V335I, *p*-value: 0.008; K800T, *p*-value: 0.01; F498L, *p*-value: 0.02; A865T, *p*-value: 0.03; G605V, *p*-value: 0.0001), two in M (p.V29A, *p*-value: 0.008; p.M44I, *p*-value: 0.04), and one in E (p.I322V, *p*-value: 0.01). Mutations that were significantly associated with mild phenotypes were present in the non-structural genes, especially in NS5. However, the mutations significantly associated with severe phenotypes are distributed in the structural region of the DENV genome. The significant non-synonymous mutations identified in clusters B and E, are summarized in Supplementary Table 7, Supplementary Figure 2.

### Effect of high frequency non-synonymous variations in E-protein and its interaction with DC-SIGN receptor

E-protein and DC-SIGN (dendritic cell-specific ICAM-grabbing non-integrin) molecular docking were performed to evaluate their potential impact on the initial degree of host invasion by the pathogen. The E-protein is essential for viral entry into host cells, as it facilitates membrane fusion during the infection process. DC-SIGN, a dendritic cell-specific receptor, is known to mediate the attachment and entry of dengue virus into cells, making it a significant target for therapeutic intervention. The specific binding site selection was based on previous research indicating that the interaction between the E-protein and DC-SIGN occurs through glycosylation sites on the E-protein, emphasizing its importance in the docking studies^39^.Three sets of mutations, corresponding to varying clinical severities, were selected from cluster A. The mutations in the E-protein are distributed as follows: in mild patients, they are located in the D1-D3 domains; in moderate, they span the D1-D3 domains and the transmembrane region; and in severe patients, they are confined to the transmembrane region.

Docking was conducted using the HADDOCK webserver, and binding energy and dissociation constants were determined using the PRODIGY tool. The binding free energies (ΔG) ranged from − 11.9 to − 13.7 kcal/mol, and the dissociation constants (K_D_) varied from 0.09 to 1 nM. The wild type exhibited the highest binding energy and lowest dissociation constant (ΔG = − 13.7 kcal/mol, K_D_ = 0.09 nM), followed by the mild (ΔG = − 13.5 kcal/mol, K_D_ = 0.1 nM), moderate (ΔG = − 12.3 kcal/mol, K_D_ = 0.9 nM), and severe, which showed the lowest binding energy and highest dissociation constant (ΔG = − 11.9 kcal/mol, K_D_ = 1.9 nM) (Table 5).

**Table 5:** Binding energies of E and DC-SIGN.

Protein–protein complex	Mutation	ΔG (kcal mol-1)	Dissociation constant (K_D_)	Interactions
Wild-type	–	− 13.7	9.20E−11	1-salt-bridge, 7-hydrogen-bond
Mild	M6I, E71,I322V	− 13.5	1.30E−10	9-hydrogen-bond
Moderate	R120T, V129I, H149N, E184D N390S, I462V, T478S	− 12.3	9.50E−10	9-hydrogen-bond
Severe	I484V	− 11.9	1.90E−09	1-salt-bridge, 6-hydrogen-bond

**Fig. 5:**
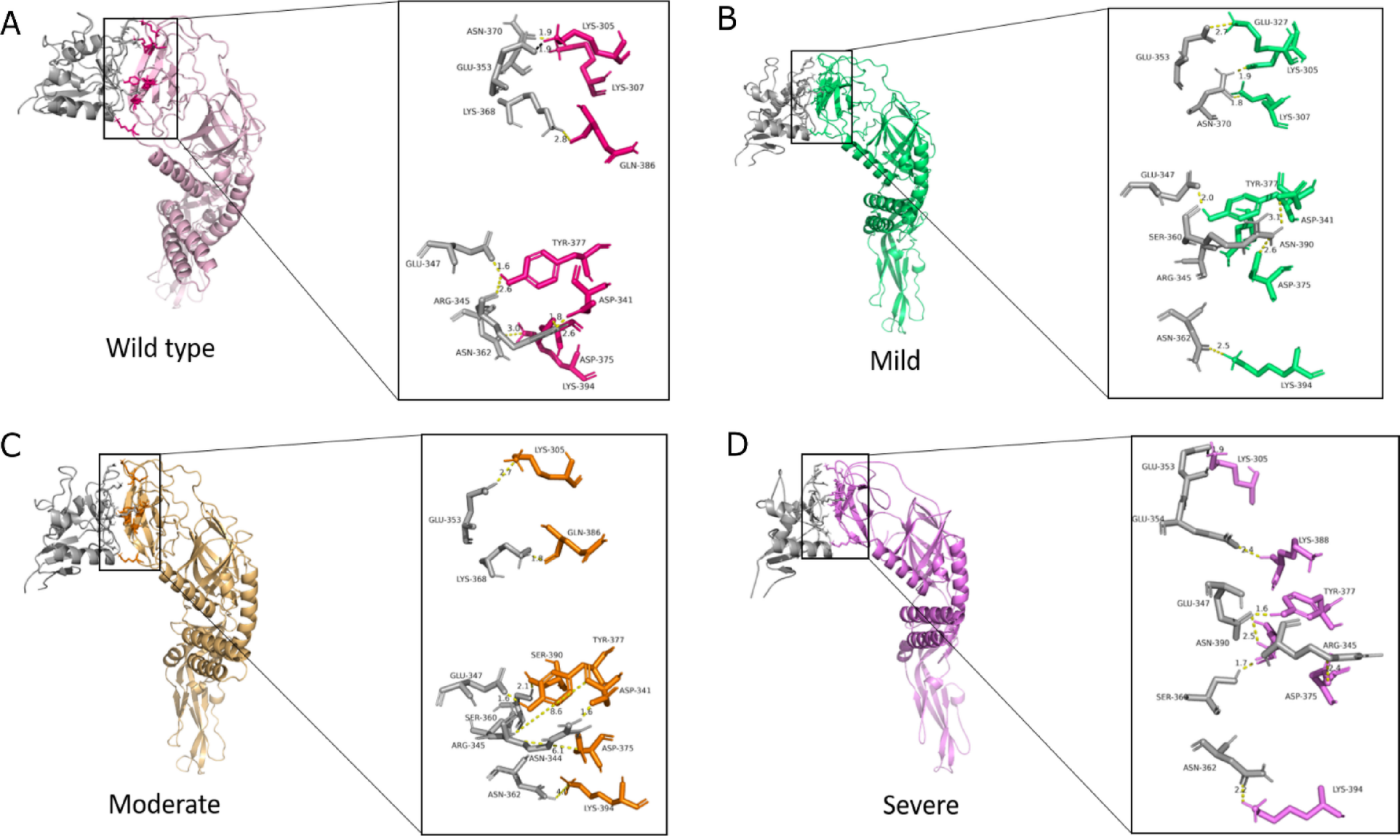
Binding and Interactions of E-protein and DC-SIGN. Binding interface of E-protein with DC-SIGN is depicted in (**A**) Wild-type, (**B**) Mild, (**C**) Moderate, and (**D**) Severe.

Further interaction analyses were performed using PDBsum and DIMPLOT from LIGPLOT to explore the types and numbers of interface residues that contribute to or diminish binding energy. It was observed that the interface residues of all complexes ranged from 15 to 18, with the severe mutation complex having the fewest E-protein interface residues and the moderate complex having the fewest DC-SIGN interface residues. Among these interface residues, there were 4–6 hydrophobic residues, with the severe patient E-protein complex exhibiting the highest number of hydrophobic residues (Fig. 5A–D). Hydrophobic residues are crucial for stabilizing protein–protein interactions^40^. Overall, hydrogen bonds were less prevalent in severe subgroup complexes compared to other protein complexes, and hydrogen bonds are vital for determining high binding efficiency^41^ (Supplementary Figure 3).

## Discussion

Several studies were conducted to study the variations in the DENV genome^42–44^. The genomic analysis in earlier studies was primarily focused on determining the genetic heterogeneity and evaluating the introduction of serotype/novel genotypes into the circulation that could be more virulent and have the potential to cause an outbreak in an endemic region. We have observed DENV-2 as a predominant serotype in the circulation. Like our study, many reports indicate that DENV-2 has consistently been the dominant serotype in recent years in India^12,45^. Moreover, a study conducted by Agarwal et al., focusing on the dengue outbreak during 2019–2021 reports all DENV serotypes in circulation with the predominance of DENV-2^46^. Additionally, like the present study, the DENV-2 was involved in all the co-infections^46^. Particularly coinciding with the dengue outbreak period of our study, DENV-2 has been also reported as a predominant serotype in circulation during 2022 in India^10^. Furthermore, we recorded the cosmopolitan as a major genotype of the DENV-2 whereas the Asian I were the minor genotype. The other studies also report observation of cosmopolitan and Asian I genotype in circulation in India^10,47^. Most importantly, unlike the present study, none of the study mentioned have conducted DENV genome wide variability analysis vis-à-vis disease severity association. The study reporting the overrepresentation of mutations in the genes are limited except that reports highlight the high mutation rate in the prM region^48^, indicating the novel insight of our analysis. The accumulation of synonymous mutations in the ancC, prM and M genes can affect viral fitness and adaptability without altering the protein structure. Additionally, dengue virus exhibits a high synonymous mutation rate, particularly beneficial for viral adaptation to different hosts (mosquitoes and humans). This adaptability is crucial for the virus’s survival and propagation, especially during periods of intense transmission^49^. Further, our analysis indicates that differential average genome coverage across the cosmopolitan genotype clustering in clade A, B and C. The mutation distribution analysis shows, clade A cover 26.8% (n = 693) unique mutations which is not shared by the cosmopolitan genotype belonging to the clade B and C. This might have attributed the clustering of those cosmopolitan genotype to the clade A. Similarly, the cosmopolitan genotype belonging to the clade B represents 20.3% (n = 523) unique mutations which are not present among the cosmopolitan genotype clustered in clade A and C (Supplementary Table 6). Based on this observation, we may speculate the differential genome coverage vis-à-vis the unique mutation shared by each clade may have attributed the clustering of cosmopolitan genotype into clade A, B and C. Moreover, since the clustering of cosmopolitan genotype into three clades is based on the DENV-2 sequence, the scope of differing these cosmopolitan genotype based on co-infecting serotype is limited.

Comprehensive analysis of CBC across the patients indicates that several parameters fall predominantly in the normal range, whereas a low count of blood parameters such as Hb, MCV, MCH, lymphocytes, and eosinophils was recorded in more than 30% of DENV infected patients. RDW was recorded high in 45.3% of patients. It is known that a low Hb and MCH, along with a high RDW count, indicate the development of anemia during DENV infection^50^. Moreover, low TLC and platelet count were noted for the 54.6% and 34.9% patients, suggesting the development of leukopenia and thrombocytopenia, respectively, whereas the hematocrit rise was recorded for the 12.8% patients. The development of leukopenia and thrombocytopenia are very frequently observed clinical manifestations in DENV infections^51^.

Interestingly, at the onset of dengue fever, the development of thrombocytopenia and leukopenia could be an early indication of the progression of DENV infection towards severity^52^. Thus, analyzing the mutation profile across the DENV-genomes vis-à-vis inferring the TLC and platelet count across the patients may be a way forward to understanding the progression of DENV infection towards severity. Moreover, it also helps to devise an early mitigation strategy for the control of the progression of DENV infection towards severity. In the present study, by employing the same approach, i.e., analysis of the mutation profile of DENV-2 genomes vis-à-vis TLC count and platelet count across the patients, we have identified a core set of mutations significantly associated with mild (normal TLC count), moderate (low TLC count and normal platelet count) and severe (low TLC and low platelet count). More precisely, we observe the association of 56 high frequency mutations to the dengue distinct disease severity ranging from mild to severe. Out of 56 high frequency mutations, we find the functions of four mutations through extensive literature survey (Supplementary Table 8). To the best of our knowledge, we are reporting additional 52 mutations for the first time which are associated with the dengue distinct severity ranging from mild to severe. Furthermore, the subsequent analysis with respect to the distribution of these significantly associated mutations across the virus genome could be an important aspect, as the presence of these significantly associated mutations in a particular genic region of the DENV genome may hold key towards understanding of the mechanistic details of virus-virulence. This may also provide a potential therapeutic target in the virus genome that could be used to control the progression of DENV infection towards severity. Our analysis, when we consider mild as a control and severe as the case, revealed that the high frequency mutations significantly associated with mild are predominantly distributed in the non-structural genes, predominantly in NS5, followed by NS4B, whereas the mutations significantly associated with severe are predominantly distributed in the structural genes. The association of these mutations with mild disease and their distribution solely in the NS5 and NS4B regions could potentially impact the virulence of the virus. It is important to note that the RdRp domain of the NS5 is a major RNA dependent RNA polymerase that facilitates the replication of the DENV-genome^53^ whereas the NS4B plays accessory role in the replication of the virus genome by interacting with other proteins needed for replication and recruiting it as a replication complex^54^. Based on the observation, it could be predicted that the high-frequency mutations, including non-synonymous mutations associated with mild symptoms, have challenged the virulence of the virus by impacting the replication of the DENV-2 genome and resulting in mild symptoms. On the other hand, the presence of mutations in the structural region has facilitated viral entry into the host, attachment, and proliferation, which could have potentially reflected severe symptoms.

To further investigate the role of non-synonymous mutations distributed in E-protein, we have adopted protein modeling and docking approaches. DC-SIGN has dual roles: mediating the pathogen into the host body^55^ and immune response activation^56^. In the survival conundrum, pathogens can develop various strategies for immune evasion by disrupting the function of dendritic cells (DCs), particularly by manipulating pattern recognition receptors (PRRs) like C-type lectin in DC-SIGN^57^. Likewise, the variations in the different disease severity establish that good binding energy and interaction increase the virus’s ability to infect the host and help it evade destruction^58,59^. In parallel, DC-SIGN also does the internalization and degradation of pathogens^60^, which explains the severe mutations having less binding compared to mild and moderate mutations.

## Limitations/future scope of the study

Present study highlights the DENV genomic variation dynamics and its association with the disease severity, it is also important to acknowledge the limitations of the study. This study includes the serum samples from the DENV infected patients to a single year outbreak in India. Studies for consecutive years would strengthen the findings. Additionally, inclusion of additional patient information such as fever onset, vomiting, headache, abdominal pain and other symptoms associated with the dengue would have strengthened further the findings presented in the manuscript. Another potential limitation of the study lies in the absence of serological data, particularly IgG and IgM levels, which are key to differentiating between primary and secondary DENV infections. Moreover, our focus was primarily on the analysis of variability across the genome of DENV-2 virus, the predominant serotype in this outbreak vis-à-vis disease severity. While the study provided deep insights into the mutational landscape of DENV-2 and its association with disease severity, the inclusion of integrative analysis of mutation across the genomes of multiple DENV serotypes (sometimes referred as coinfection of DENV serotypes) and clinical data would broaden the scope in future studies*.*

## Electronic supplementary material

Below is the link to the electronic supplementary material.

Supplementary Material 1

Supplementary Material 2

Supplementary Material 3

Supplementary Material 4

Supplementary Material 5

Supplementary Material 6

Supplementary Material 7

Supplementary Material 8

Supplementary Material 9

## Data Availability

The DENV-2 genomic data has been uploaded to GISAID (IDs available in Supplementary Table 2).
