# Supplementary material for: Clinico-genomic study reveals association of dengue virus genome high frequency mutations with dengue disease severity
Source: Sci Rep. 2025 May 28;15:18724. doi: 10.1038/s41598-025-00462-z (PMC12120001; doi:10.1038/s41598-025-00462-z)
Supplement: Supplementary file 1 — Supplementary Material 1 [file 41598_2025_462_MOESM1_ESM.pdf]

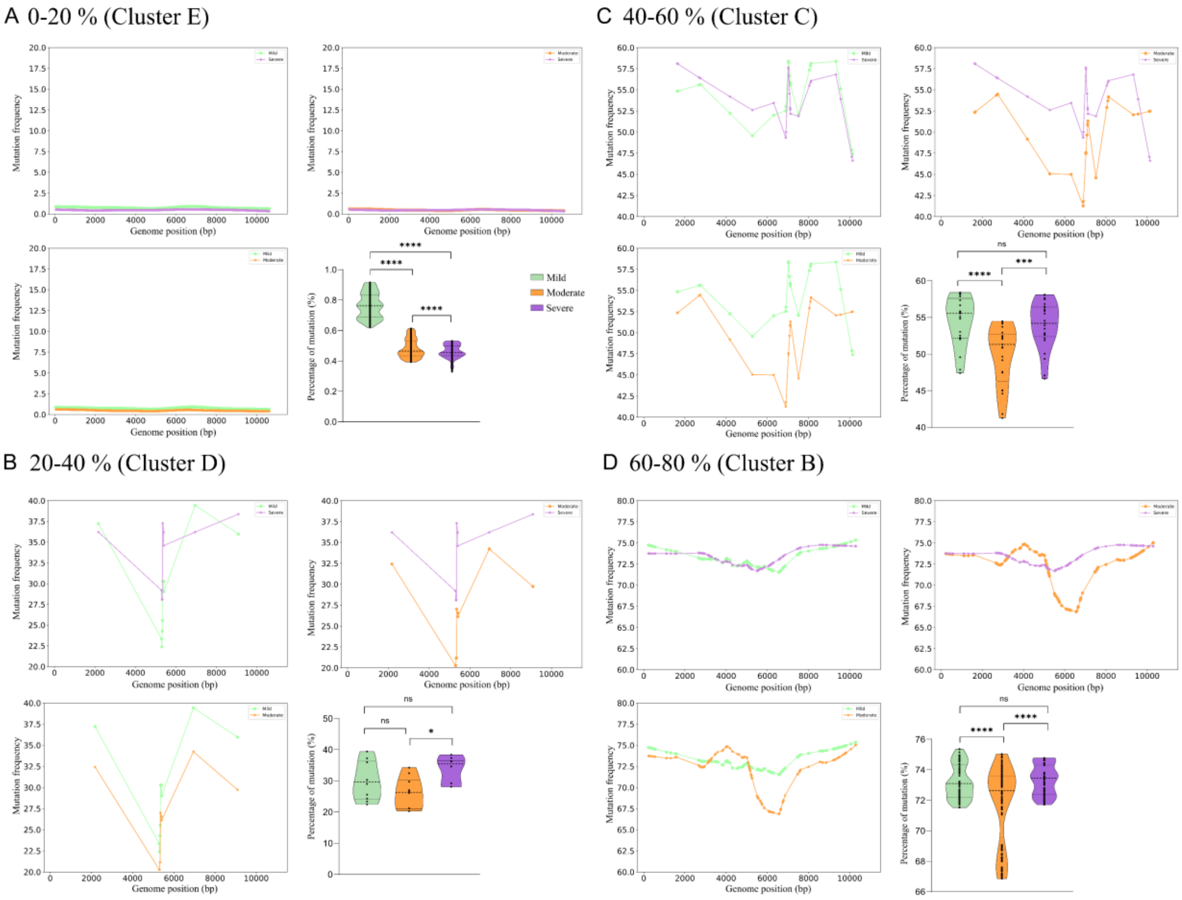

Supplementary Figure 1: (A-D) Line plots showing mutation frequency across the DENV-2. genome for Cluster B-E and Violin plot showing significant differences between percentage of mutation and severity cohort.

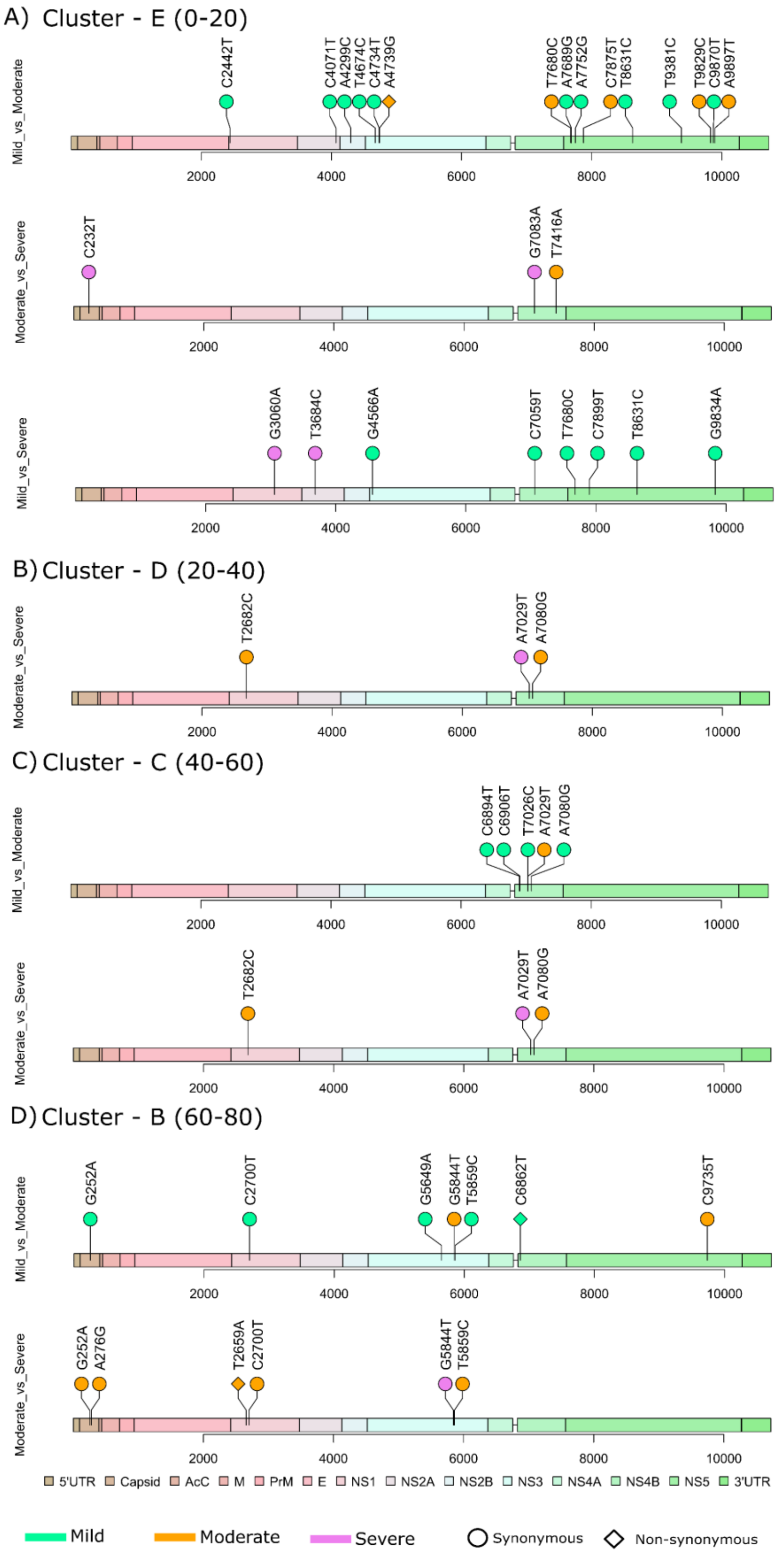

Supplementary Figure 2: (A-D) Lollipop plot representing significant synonymous and non-synonymous mutations between severity groups.

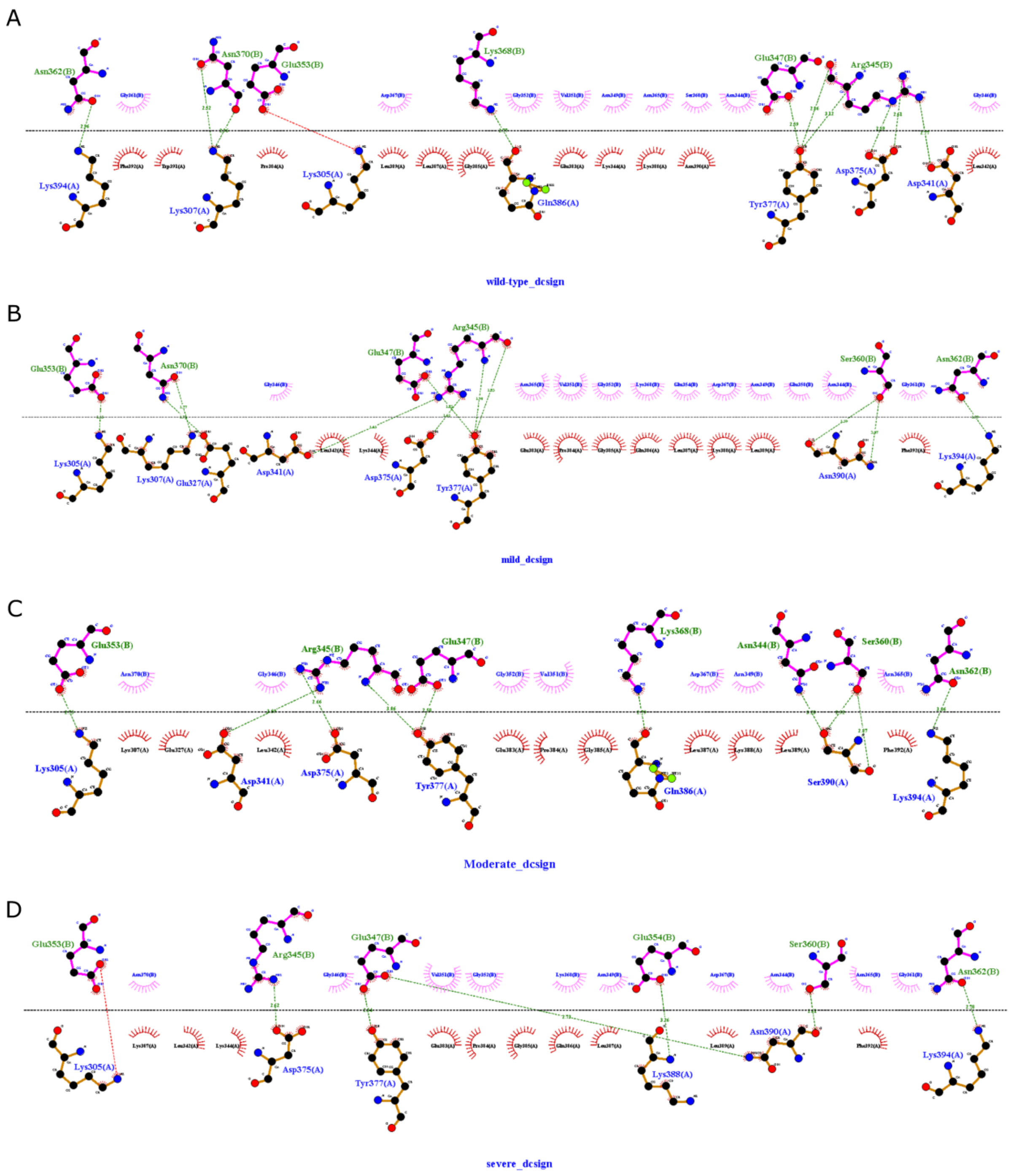

Supplementary Figure 3: (A-D) Interaction plot between E-protein and DC-SIGN.
